# A theoretical study of the time-resolved x-ray absorption spectrum of the photoionized BT-1T cation

**DOI:** 10.1063/4.0000183

**Published:** 2023-05-26

**Authors:** Anna Kristina Schnack-Petersen, Mátyás Pápai, Sonia Coriani, Klaus Braagaard Møller

**Affiliations:** 1 Department of Chemistry, Technical University of Denmark, DK-2800 Kongens Lyngby, Denmark; 2 Wigner Research Centre for Physics, P.O. Box 49, H-1525 Budapest, Hungary

## Abstract

The time-resolved x-ray absorption spectrum of the BT-1T cation (BT-1T^+^) is theoretically simulated in order to investigate the charge transfer reaction of the system. We employ both trajectory surface hopping and quantum dynamics to simulate the structural evolution over time and the changes in the state populations. To compute the static x-ray absorption spectra (XAS) of the ground and excited states, we apply both the time-dependent density functional theory and the coupled cluster singles and doubles method. The results obtained are in good agreement between the methods. It is, furthermore, found that the small structural changes that occur during the reaction have little effect on the static XAS. Hence, the tr-XAS can be computed based on the state populations determined from a nuclear dynamics simulation and *one set* of static XAS calculations, utilizing the ground state optimized geometry. This approach can save considerable computational resources, as the static spectra need not to be calculated for all geometries. As BT-1T is a relatively rigid molecule, the outlined approach should only be considered when investigating non-radiative decay processes in the vicinity of the Franck–Condon point.

## I. INTRODUCTION

**FIG. 1.**
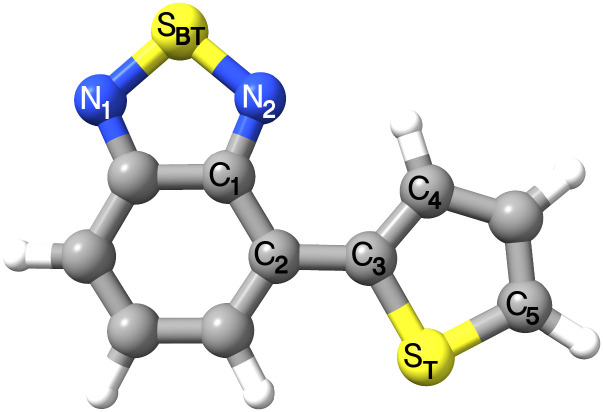
Structure of the BT-1T molecule optimized on *S*_0_ at the BHHLYP/cc-pVDZ level of theory. The atoms important for the structural parameters considered are labeled. The benzothiadiazole unit is seen on the left, while the thiophene unit is the right part of the molecule.

The molecule 4-(2-thienyl)-2,1,3-benzothiadiazole (here denoted BT-1T) consists of an electron-accepting benzothiadiazole unit and an electron-donating thiophene unit (see Fig. 1). Several *π*-conjugated polymers, including either one or both of these units, have been scrutinized as potential organic solar cells^1–4^ and generally for organic electronic devices.^5,6^ Particularly, applications in organic solar cells are of interest. Such solar cells are becoming a feasible alternative to the more established silicon- or metal-based ones due to their lower production costs^7,8^ and with organic solar cells now reaching efficiencies of around 18%.^9–12^ An important property of an organic solar cell is its ability to form charge transfer states.^13,14^ Thus, this process must be investigated for any potential new solar cell in order to determine its suitability.^13^

Although molecules including BT-1T units have already been investigated both experimentally and theoretically,^15,16^ the small BT-1T molecule has only recently been studied theoretically on its own by Khalili *et al.*, who considered not only the photoexcitation^17^ but also the photoionization of the system.^18^ So far, experimental studies of this system have not been published. Since experimental studies do typically rely on theoretical support for a full interpretation of the often complicated experimental spectra,^19–22^ further theoretical studies are, nonetheless, still required, especially at higher levels of theory.

**FIG. 2.**
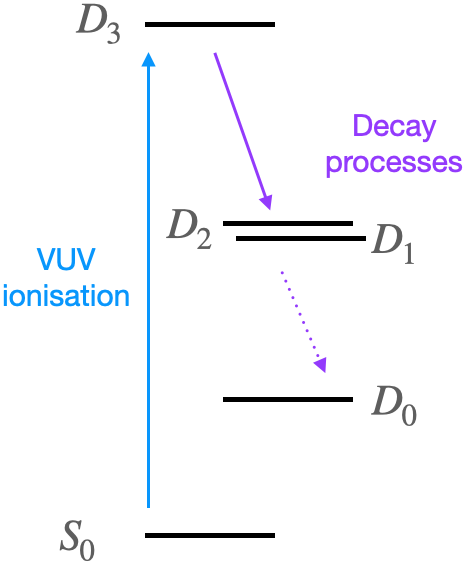
Schematic illustration of the studied process, where the molecule is photoexcited to the *D*_3_ state. The system subsequently deexcites to the *D*_2_ and *D*_1_ states, while the *D*_0_ state does not become significantly populated within the time studied here.

**FIG. 3.**
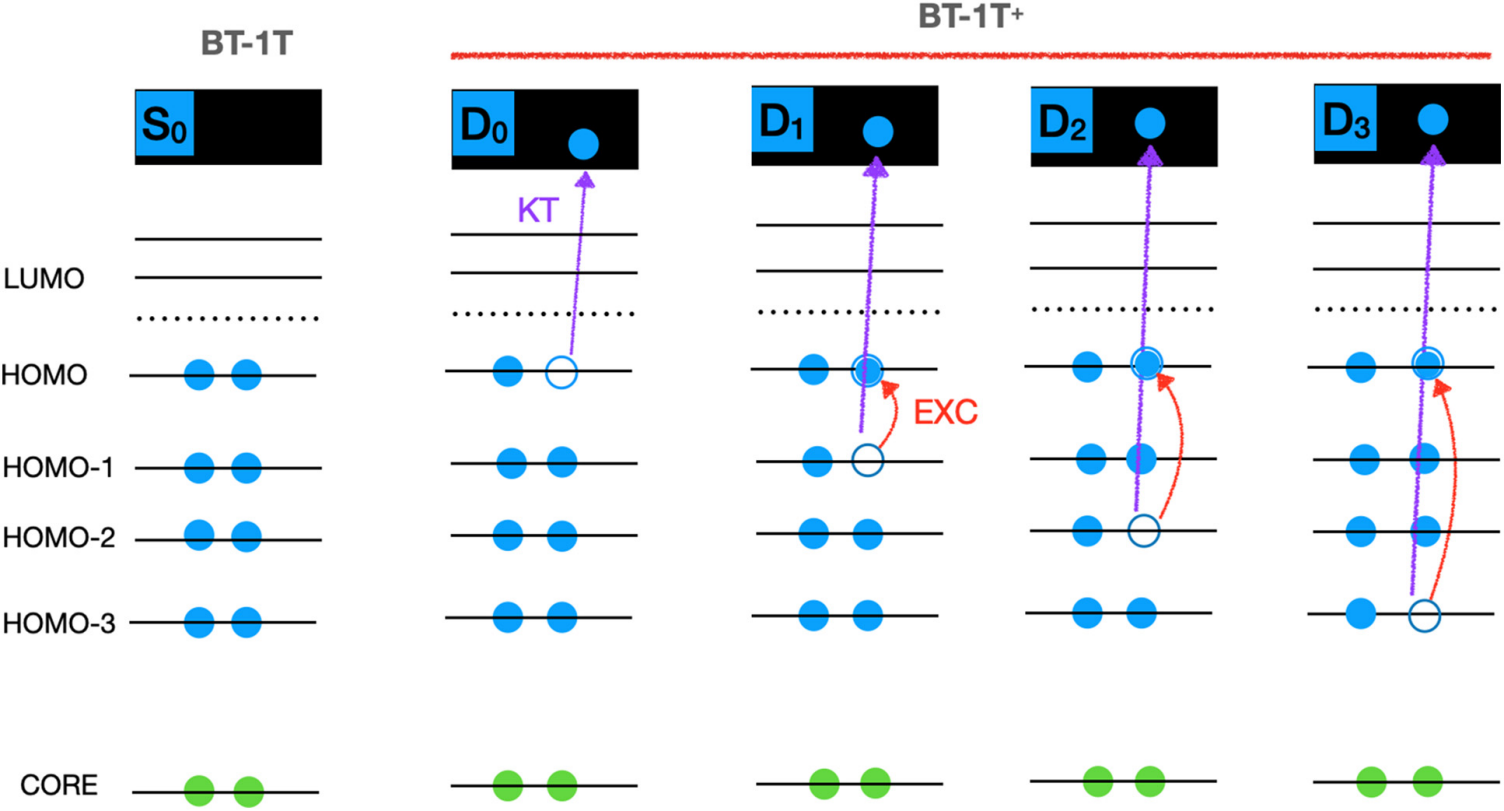
Molecular orbital schematic representation of how the relevant states of BT-1T^+^ are obtained in the present study (excitations from the ground state *D*_0_ of the cation, red arrows) and by Khalili *et al.*^18^ (KT valence ionization from the *S*_0_ ground state of the neutral molecule, blue arrows).

Khalili *et al.*^17,18^ studied the BT-1T unit by simulating its time-resolved x-ray absorption spectra (tr-XAS). X-ray spectroscopy and scattering offer time-resolutions on the sub-picosecond timescale characteristic of molecular reactions.^23–28^ Furthermore, XAS, especially excited state XAS, has proven sensitive to both electronic and structural changes in small organic molecules.^22,29,30^ In Ref. 18, the authors explored, in particular, the possibility to use tr-XAS to follow the charge dynamics of the BT-1T system after photoionization with a vacuum ultraviolet (VUV) pulse. They prepared the BT-1T^+^ cation in a state corresponding to the removal of one of the HOMO-3 electrons in the neutral molecule (see Figs. 2 and 3).

Khalili *et al.*^18^ employed Hartree–Fock (HF) level calculations using a small basis set, which is a rather low level of theory; calculations at a higher level of theory are, therefore, needed to determine the validity of the results. Moreover, the dynamics of the process was studied with trajectory surface hopping (TSH), which treats the non-adiabatic process stochastically. While this is often a good approximation, it is also of interest to investigate the effect of using quantum dynamics. We will, therefore, study the BT-1T system quantum dynamically for the process of tr-XAS (probe step) following the VUV photoionization (pump step) of the neutral species. We will investigate not only whether this type of experiment is suitable for studying the important charge transfer reaction, but also how much the conclusions are affected by resorting to a higher level of theory. Khalili *et al.*^18^ employed ionic potential energy surfaces (PESs) based on Koopmans' theorem (KT). Instead, we will investigate the nuclear dynamics by applying time-dependent density functional theory (TDDFT) directly to the cation. Unlike HF, TDDFT includes some electron correlation,^31^ without increasing the computational cost beyond feasibility. In general, TDDFT has proven a very successful method due to its low computational cost and overall good performance,^32–34^ even though the choice of functional can greatly affect the results, and its effect is often difficult to predict.^35,36^ Figure 3 pictorially illustrates the differences between the approach used by Khalili *et al.*^18^ and the one used in the present study when considering the molecular orbitals of the neutral molecule.

Following the nuclear dynamics simulations, XAS calculations are then carried out utilizing TDDFT as well as frozen core-valence separated equations-of-motion coupled cluster singles and doubles (fc-CVS-EOM-CCSD).^37^ This latter method is computationally more demanding, but the quality of the results obtained with CC methods is more predictable and can be systematically improved. Indeed, it is in the CC family of methods that the gold standard for calculations is to be found when the ground state is dominated by a single configuration.^38–40^ While not being the gold standard, CCSD still yields results of relatively high quality and has become one of the methods of choice for accurately computing molecular properties at a reasonable cost.^40^

The paper is organized as follows. Section II collects all information on the computational approaches utilized for this study. In Sec. III, we present the relevant molecular orbitals (MOs) and valence transitions within the cation, while in Sec. IV, we report the calculated charges on both the sulfur atoms and the two units of the molecule in the electronic states of interest. Next, the dynamics simulation is presented in Sec. V. Here, we perform a TSH simulation^41^ for a limited number of trajectories in order to determine the normal modes to be included in a quantum dynamics simulation using the multi-configurational time-dependent Hartree (MCTDH) method.^42,43^ The results of these simulations are compared and evaluated, before the XAS calculations are considered in Sec. VI. To simulate the tr-XAS, the predicted average geometries and state populations from the MCTDH simulation are used in Sec. VI C. Here, both the importance of the geometrical changes, which are expected to be small due to the rigidity of the molecule, and the electronic state are evaluated.

## II. COMPUTATIONAL METHODS

*Geometries*: The molecular geometry of the molecule before ionization was determined by optimizing the molecular structure of the neutral species on the ground state using the ORCA-5.0.0 program package.^44,45^ The optimization was carried out at the DFT level of theory employing the BHHLYP functional and the cc-pVDZ basis set.^46,47^

Since the photoionization, starting the studied process, is fast, the initial geometry of interest (at a time delay of zero fs) is that of the neutral species. The geometries for non-zero time delays are determined as the average structures (i.e., centroid geometries) found in the quantum dynamics simulation.

*Dynamics*: For all calculations related to the dynamics simulations, the (TD)DFT/BHHLYP/cc-pVDZ level of theory was used. For excited state calculations, the Tamm–Dancoff approximation (TDA) was employed. This approach has been used previously with good results.^48^

First, the normal modes were determined analytically for the optimized structure. To determine the relevant normal modes to include in the MCTDH calculation, a TSH calculation was carried out in the SHARC-2.1.1 program package,^41,49,50^ interfaced with ORCA-4.2.0.^44,51^ The older version of ORCA was utilized here, since the auxiliary python-scripts necessary to perform the SHARC calculation were not adapted to the newer version. In the TSH simulation, 25 trajectories were simulated for 400 fs. This number of trajectories is clearly too small to obtain good statistics for predicting quantum yields and lifetimes. It has, however, previously been shown that a limited number of trajectories can qualitatively determine the normal modes of interest^48^ as long as one is not concerned with rare events. The TSH simulation was carried out from a Wigner distribution of geometries and photoexcitation of the cation to simulate initial conditions. The final states considered are the same with respect to the occupation of neutral MOs, as those obtained by a photoionization of the neutral species (see Fig. 3). The gradient in all states, and hence the PES points, is like the initial conditions, generated by considering photoexcitation of the ion. This way of generating the PES is employed even if initial conditions are chosen based on photoionization of the neutral species (such a simulation can be found in the supplementary material, Sec. S3.2). The (adiabatic) PES in the TSH simulation was determined on the fly. The simulation started in the third excited state (spin state 7 in the diagonal representation). The calculation included spin–orbit couplings and was, therefore, performed with SHARC dynamics,^52^ i.e., based on the diagonal representation of the Hamiltonian. This is an alternative to the molecular Coulomb Hamiltonian (MCH) representation,^52^ which is known as regular surface hopping. Couplings between states were approximated based on wavefunction overlaps, and an energy based decoherence correction was employed.

The MCTDH simulation was carried out along selected normal modes of interest, determined based on the TSH simulation (see Sec. V B and Sec. S3.1 in the supplementary material). The (diabatic) PESs for the MCTDH calculation were fitted to a vibronic-coupling Hamiltonian^53,54^ with a harmonic oscillator zero-order term and dimensionless mass-frequency scaled normal coordinates. Harmonic oscillator eigenfunctions were used as a basis for the PES fit. The fit for each normal mode was based on 31 points along the given mode for displacements *Q* = −15 to *Q* = 15, as some modes showed displacements of |Q|>10. The fitting parameters are given in the supplementary material (Sec. S3.4). All necessary points were calculated with ORCA-5.0.0, and the quantum dynamics simulation was then carried out with the MCTDH-8.4.12 program package.^55^ Input for the latter program was based on a local development version of the VCHAM program,^56^ modified to accommodate output files generated by ORCA-5.0.0.

The MCTDH calculation employed a primitive basis consisting of 45 basis functions for each normal mode chosen. The *D*_0_ state for each mode was described by five single particle functions, while 10 functions were used to describe the remaining states for each mode. The grid populations were investigated and found to be below $10^{-5}$, indicating a converged primitive basis. Likewise, the natural weights were investigated to ensure convergence with respect to the single particle functions. Again, sufficiently small numbers were found (of the orders of magnitude: $10^{-7}$– $10^{-3}$). In addition, multimode single particle functions were used to combine modes in order to reduce computational costs.

*Charges*: Calculations of CHELPG (CHarges from ELectrostatic Potentials using a Grid based method^57^) point charges were performed in Gaussian 16^58^ at the BHHLYP/cc-pVDZ level of theory, in order to obtain the excited state charges. Such CHELPG charges have previously been successful in applications for classical nuclear dynamics.^59^

*X-ray absorption spectra*: The XAS were calculated at both the TDDFT level of theory with the BHHLYP functional and at the fc-CVS-EOM-CCSD level of theory, adopting the aug-cc-pVTZ basis on sulfur and cc-pVDZ on the remaining atoms. Q-Chem-5.4.2^60^ was used for these calculations.

The XAS spectrum of each electronic state of the cation was determined using TDDFT/fc-CVS-EOM-CCSD. These calculations were based on the Kohn–Sham/CCSD wavefunction of the given state optimized by employing the initial maximum overlap method^61,62^ (IMOM) procedure. Here, the excited state wavefunction is generated by solving the self-consistent field equations, imposing as the constraint that the occupied orbitals have the largest possible overlap with an initial set of guess orbitals, rather than utilizing the Aufbau principle. This strategy has previously been employed with success in a number of cases.^30,63–65^ In our calculations, the choice of initial guess was based on the orbitals determined for the ground state of the neutral molecule. The occupied orbitals were then specified to be the same with one exception, since the cation has a singly occupied orbital. The choice of this singly occupied orbital for each investigated state is discussed in Sec. III and can also be anticipated based on Fig. 3. The IMOM approach has the disadvantage of generating excited states that are not orthogonal to the ground state. On the other hand, the XAS of the excited states can be straightforwardly computed as one-photon absorption of the IMOM-optimized state. Thus, one can obtain not only the core to singly occupied MO (SOMO) transitions but also transitions from core orbitals to virtual MOs. An alternative approach for simulating excited state XAS is to compute the intensities as the transition strengths between two excited states, namely, the initial valence excited and the final core excited state. The corresponding transition energies are then computed as energy differences between the excitation energies of the two states from the ground state. At the CCSD level, this “quadratic response” approach can, however, only reliably describe core-to-SOMO transitions, as only these correspond to a single photon process from the ground state. An equivalent approach for TDDFT is currently not available in Q-Chem-5.4.2.

A default convergence criterion of 10^−6^ was used for the excitation energy TDDFT calculations, while a convergence threshold of 10^−5^ was applied for the computationally more demanding EOM-CCSD calculations. All calculations on the cation employed an unrestricted formalism. TDA was not used for these calculations.

*Orbitals*: Ground state MOs are plotted based on Gaussian 16 calculations. Excited state MOs were, however, found by utilizing the Q-Chem-5.4.2 program package. All natural transition orbitals (NTOs) were determined based on calculations in Q-Chem-5.4.2. NTOs and MOs were plotted using Molden^66^ and a contour value of 0.01.

## III. ORBITALS OF INTEREST

**FIG. 4.**
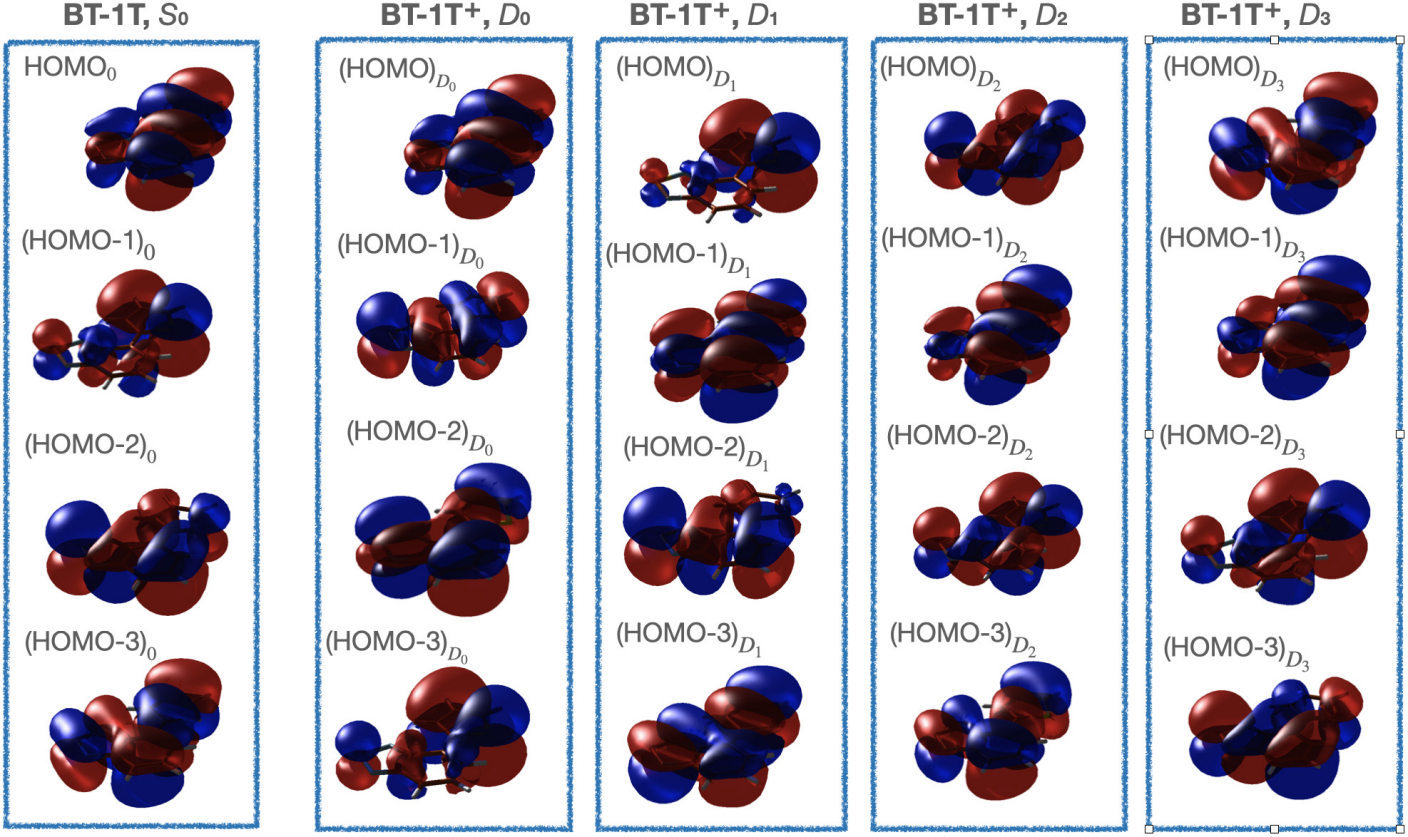
Highest-lying occupied MOs in BT-1T and in BT-1T^+^ in its ground state (*D*_0_) and first three excited states (*D*_1_, *D*_2_, and *D*_3_). The MOs of *S*_0_ were obtained at the BHHLYP/cc-pVDZ level of theory; those of the cation are BHHLYP results with the aug-cc-pVTZ basis on sulfur and the cc-pVDZ basis on the remaining atoms. Note the similarity between ${\left(\text{HOMO}\right)}_{D_{n}}$ and (HOMO-n)_0_.

The MOs of interest are the HOMO, HOMO-1, HOMO-2, and HOMO-3 displayed in Fig. 4 for both the neutral molecule and the cation. The obtained MOs for the neutral molecule are in good agreement with those presented by Khalili *et al.*^17,18^ The MOs of the cation will be discussed further below. Throughout the text, we will refer to the orbitals for the neutral molecule with a subscript “0” and to those for the cation with a *D_n_* subscript, indicating the considered state. Hence, the HOMO for the neutral molecule is denoted HOMO_0_.

To generate the wavefunction of the cation, a *β*-spin electron was removed from the neutral molecule. Removal of an electron from the HOMO results in a slightly spin contaminated *D*_0_ state with $\langle S^{2}\rangle =0.8163$ at the BHHLYP/cc-pVDZ level of theory (a pure doublet spin state has $\langle S^{2}\rangle =0.75$).

**FIG. 5.**
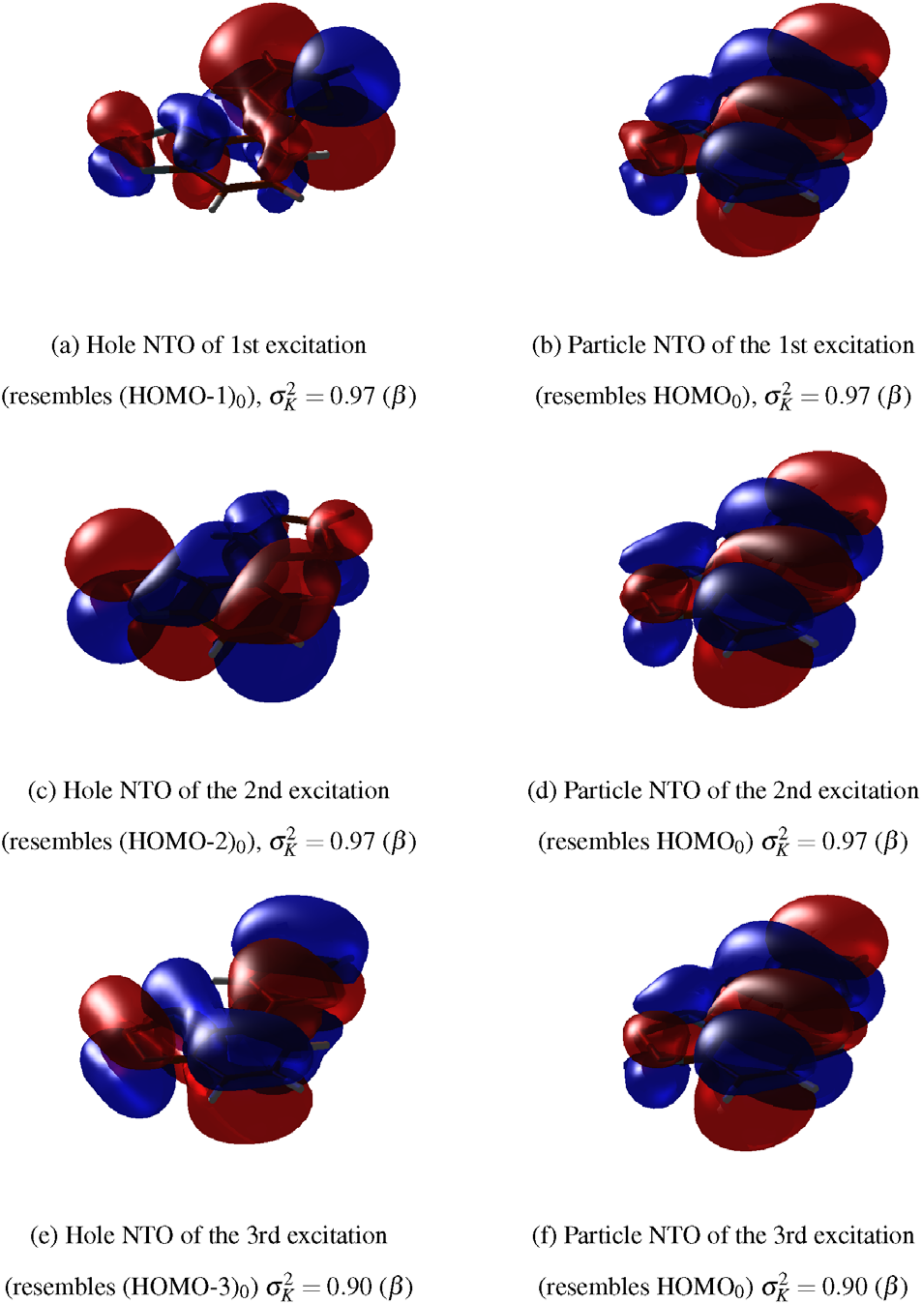
The dominating NTOs for the three lowest lying transitions in BT1T^+^ at the minimum-energy structure of the neutral molecule calculated at the TDA/BHHLYP/cc-pVDZ level of theory. The orbital of origin for the excited electron is denoted as hole, while the final orbital is denoted as particle. $\sigma_{K}^{2}$ indicates the weight of the NTO pair, while the spin of the NTO is given in parenthesis. (a) Hole NTO of first excitation, (b) particle NTO of the first excitation, (c) hole NTO of the second excitation, (d) particle NTO of the second excitation, (e) hole NTO of the third excitation, and (f) particle NTO of the third excitation.

Taking the *D*_0_ state of the cation as the reference, we also computed the NTOs for its first three valence transitions, which are shown in Fig. 5. These NTOs closely resemble the MOs shown in Fig. 4 (left). The particle NTO for all valence excitations corresponds to HOMO_0_, which must, therefore, be singly occupied in the cationic ground state denoted *D*_0_. *D*_0_ corresponds to the hole in HOMO_0_, *D*_1_ corresponds to the hole in (HOMO-1)_0_, *D*_2_ corresponds to the hole in (HOMO-2)_0_, and, finally, *D*_3_ corresponds to the hole in (HOMO-3)_0_. For consistency, we shall throughout denote the state associated with a hole in the (HOMO-n)_0 _MO as *D_n_*.

The MOs and NTOs determined by using the methods and basis sets employed to calculate the XAS are found to agree well with the MOs and NTOs shown here (see the supplementary material, Secs. S2.2 and S2.3).

The four highest-energy occupied MOs of the cation, in each of the four different electronic states considered in the XAS calculations, are shown in columns 2 to 4 of Fig. 4. We only explicitly generate the states *D*_1_-*D*_3_ when calculating the XAS. Therefore, the MOs of the cation are shown for the basis sets also employed in these calculations, i.e., the aug-cc-pVTZ basis on sulfur and cc-pVDZ on the rest. Note that the Unrestricted Hartree–Fock (UHF) MOs used in the CC calculations on the cation (Sec. S2.3) are in good agreement with the ones shown here. Furthermore, the MOs of *D*_0_ clearly resemble those obtained at the BHHLYP/cc-pVDZ level of theory (see Sec. S2.1). We highlight the different energetic ordering of the MO orbitals of equivalent character in the neutral molecule and in each of the four states of the cation. For these cationic states, the orbital with the highest energy (the ${\text{HOMO}}_{D_{n}}$) is always the singly occupied one, which corresponds to one of the four highest occupied MOs of the neutral molecule [the (HOMO-n)_0_]. In short, the SOMO is in all cases ${\text{HOMO}}_{D_{n}}$, which has the same character as (HOMO-n)_0_.

Based on the characters of the MOs obtained for *D*_0_ (see Fig. 4), we note that generating, e.g., *D*_1_ corresponds to a hole in ${\left(\text{HOMO}‐3\right)}_{D_{0}}$. We inspected the weights of the different transitions contributing to the *D*_1_ excitation and found that the main contribution to this excitation is, indeed, from the transition originating in ${\left(\text{HOMO}‐3\right)}_{D_{0}}$ and with final state in ${\text{HOMO}}_{D_{0}}$. We did, however, note from these weights that each state cannot be associated with only one transition (although one is found to dominate in each case). This might partly explain why the *D*_1_ state is associated with a transition originating in the ${\left(\text{HOMO}‐3\right)}_{D_{0}}$. Despite the lower energy of the ${\left(\text{HOMO}‐3\right)}_{D_{0}}$ MO compared to ${\left(\text{HOMO}‐1\right)}_{D_{0}}$, the total energies of the *D_n_* states increase as expected from *D*_0_ to *D*_3_ (see Table III). As previously mentioned, *D_n_* is here generated by moving one electron from the MO with the character of (HOMO-n)_0_ to that with the character of HOMO_0_. Thus, our chosen naming convention of the states is also in line with the energetic ordering at the ground state optimized structure.

The reordering of MOs indicates that we cannot apply the simple picture of the MOs retaining a predetermined ordering upon ionization, which is generally assumed, e.g., when using Koopmans' theorem. Such reordering of the orbitals upon excitation has also been observed in a recent study by Schmerwitz *et al.*^67^ The final states obtained in our approach should, however, be noted to correspond well with what would also be expected based on Koopmans' theorem. Our findings do indicate, though, that analyzing the process in terms of one electron moving stepwise down through the MOs might be too simple.

## IV. CHARGE CALCULATIONS

In order to investigate the charge transfer reaction, we computed the CHELPG point charges of the cation in the ground state as well as in the three lowest lying excited states (see Table I). We observe only small changes in the charge on the sulfur atom located in the benzothiadiazole unit (labeled S_*BT*_) across the considered states. A large change is, however, observed on the sulfur atom in the thiophene unit (labeled S_*T*_) in *D*_1_. We note that electron density is, thus, moved away from S_*T*_ in *D*_1_, as was also concluded by Khalili *et al.*^18^ One could, therefore, in principle, observe the migration of electron density away from S_*T*_ by monitoring the de-excitation process from *D*_3_ to *D*_1_. Another observable indication of the electron density migration might be the strength of the core transitions originating on the different sulfur atoms, since a high intensity is generally associated with high electron density on the atom on which the core transition originates. This will be further considered in Sec. VI C. It should be noted, however, that the changes in charge density on S_*BT*_ do not correspond to the changes of the charge density on the entire benzothiadiazole unit, although a good qualitative agreement is observed for the sulfur atom in the thiophene unit. While the charge density on S_*BT*_, as mentioned, does not display any significant changes, it is clear that negative charge is moved from the thiophene unit to the benzothiadiazole unit from *D*_3_ to *D*_1_. Hence, while S_*T*_ might be the most important atom to consider for the charge transfer in the thiophene unit, one cannot simply inspect the S_*BT*_ atom of the benzothiadiazole unit. Instead, the entire unit should be scrutinized.

**TABLE I.** CHELPG charges of the sulfur atoms of BT-1T^+^ as well as of the benzothiadiazole and thiophene units in the ground state (*D*_0_) and in the three lowest excited states (*D*_1_, *D*_2_, and *D*_3_) calculated at the BHHLYP/cc-pVDZ level of theory.

	S_*BT*_	S_*T*_	*BT*-unit	*T*-unit
D3	0.5741	0.0403	0.7013	0.2987
D2	0.5034	0.0156	0.8039	0.1961
D1	0.4324	0.4124	0.3083	0.6917
D0	0.4503	0.0233	0.5710	0.4290

## V. DYNAMICS SIMULATION

When performing the MCTDH calculation (as well as the TSH simulation), the PESs are calculated based on excited state energies of the cation. The starting point of the simulation is the *D*_3_ excited state. The zero point energy of the calculation is set to the ground state energy of the neutral molecule at its equilibrium geometry, as this is the starting point of the corresponding experiment.

The normal modes that appear to be of interest in the TSH simulation are evaluated further in order to choose the modes to be included in the MCTDH calculation (see the supplementary material, Secs. S3.1–S3.3).

### A. Population analysis of TSH results

The evolution of populations in the TSH simulation is studied by considering the states based on energy ordering (i.e., adiabatic states), which is the natural choice for TSH simulations. It is also possible to consider the states based on oscillator strengths. The latter can be useful for excited states that are close in energy but have oscillator strengths of different magnitudes. This is the case here (see Table II), however, the oscillator strengths are seen to change significantly during the reaction (cf. Tables S3, S20, and S21). Hence, it is difficult to define the states in terms of non-overlapping oscillator strength intervals. This approach has, thus, been deemed inappropriate for the system considered here. The populations based on energies are determined by the fraction of trajectories in each state at a given time. Moreover, to better compare the evolution of the populations determined here to the one determined using MCTDH, the populations are also transformed to a diabatic basis using auxiliary SHARC scripts. These rely on overlap matrices as well as wavefunction coefficients in the diabatic basis.

**TABLE II.** Excitation energies (eV) and oscillator strengths $f_{\text{osc}}$ from *D*_0_ to the excited states *D*_1_–*D*_3_ calculated at the TDA/BHHLYP/cc-pVDZ level of theory at the ground state optimized structure of the neutral molecule.

Excited state	Excitation energy (eV)	$f_{\text{osc}}$
*D*_1_	1.669	0.000 631 580
*D*_2_	1.967	0.017 129 803
*D*_3_	2.100	0.220 979 269

When simulating XAS later on, one must be careful to verify whether the first and second excited states do, indeed, correspond to a hole in (HOMO-1)_0_ and (HOMO-2)_0_, respectively. At the geometries associated with our investigated time delays, the NTOs for the three valence excitations appear unchanged compared to the NTOs at the initial geometry (see Figs. S3, S46, and S47). This indicates that we can associate the *D_n_* state with a hole in (HOMO-n)_0_ throughout.

**FIG. 6.**
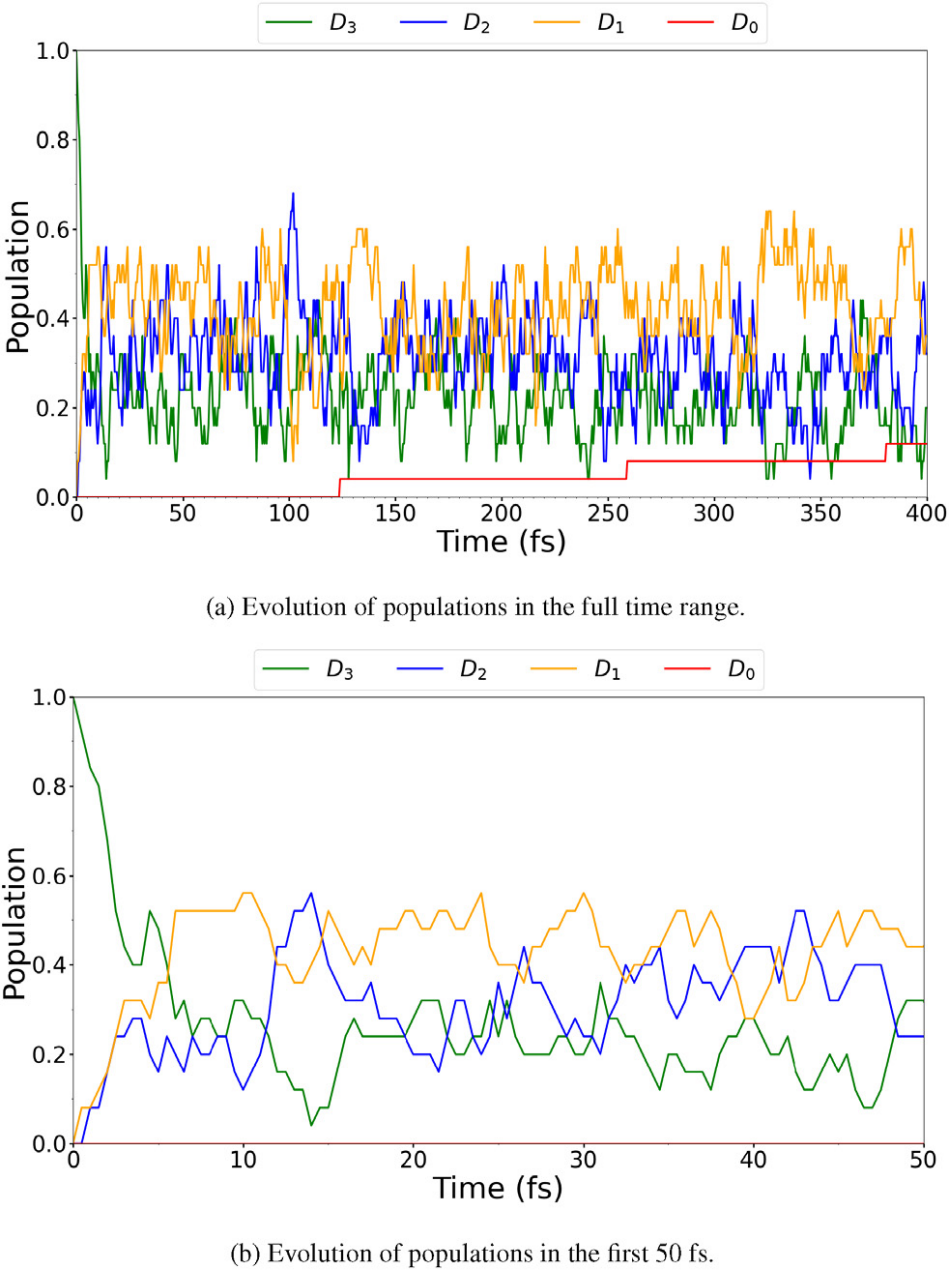
BT-1T^+^: Evolution of classical (adiabatic) populations of *D*_0_, *D*_1_, *D*_2_, and *D*_3_ in TSH using SHARC dynamics based on energies plotted over the entire simulated time range (a) and over the first 50 fs (b). Populations are calculated based on an auxiliary SHARC script.

**FIG. 7.**
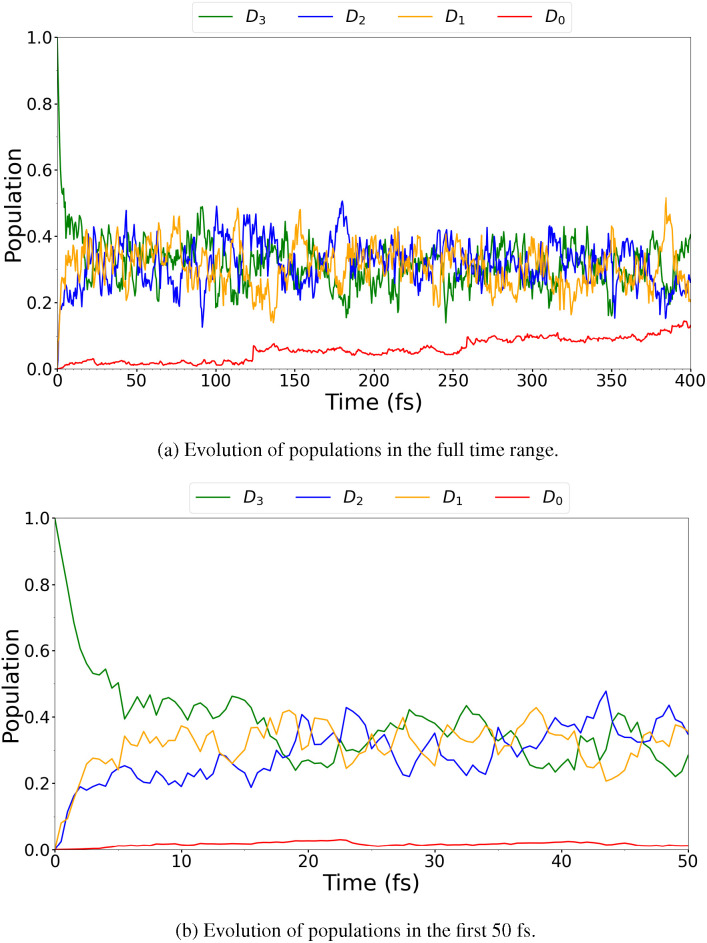
BT-1T^+^: Evolution of the populations of *D*_0_, *D*_1_, *D*_2_, and *D*_3_ in TSH using SHARC dynamics based on a transformation to the diabatic basis over the entire simulated time range (a) and over the first 50 fs (b). Populations are calculated based on an auxiliary SHARC script.

The TSH simulation gave rise to three trajectories jumping to the *D*_0_ state. Plots of the evolution of the populations can be seen in Fig. 6. The diabatized populations were also determined using an auxiliary SHARC script, and the results are shown in the plots in Fig. 7. The agreement between Figs. 6 and 7 is excellent, although Fig. 7 shows a smoother evolution of the populations compared to Fig. 6. This result indicates that, in this case, the diabatic and adiabatic pictures give rise to the same states, further justifying our choice of denoting the different states as *D_n_* for an empty (HOMO-n)_0 _MO throughout.

The TSH simulation shown here exhibits the same rapid decrease in the *D*_3_ population and simultaneous increase in the populations of *D*_2_ and *D*_1_ as also shown by Khalili *et al.*^18^ We, however, obtain a significantly lower yield of *D*_0_ compared to the previous study. This discrepancy could be a result of the different levels of theory used. Furthermore, the wavefunction of the ionized species was determined by Khalili *et al.*^18^ based on Koopmans' theorem,^68,69^ without including additional relaxation. The otherwise good agreement leads us to conclude that the normal modes of interest for the reaction at this level of theory can, indeed, be determined based on the presented TSH simulation. We also note that the evolution of the populations appears very similar in the two additional TSH simulations presented in the supplementary material, Secs. S3.2 and S3.3. In Sec. S3.2, photoionization is used to generate initial conditions, while in Sec. S3.3, the nuclear dynamics was carried out in the MCH representation for initial conditions generated based on photoexcitation of the cation. This indicates that the results presented are relatively robust with respect to small changes in the initial condition generation and the state representation, in which the nuclear dynamics is considered.

### B. Determining the normal modes for an MCTDH simulation

When determining which normal modes to include in an MCTDH calculation based on a TSH simulation, many types of analyses can be made;^70^ one can consider the activity of the modes as well as coherence and correlation. The linear vibronic-coupling (LVC) parameters can also be investigated. Based on all these types of analyses (see the supplementary material, Sec. S3.1), ten normal modes of interest were identified: modes 1, 2, 8, 16, 24, 33, 44, 46, 47, and 48 (labeled based on frequencies of increasing magnitude). These ten modes were also found to be of importance in the two additional TSH simulations shown in Secs. S3.2 and S3.3.

### C. Analysis of the generated PES for normal modes of interest

The PESs of the ten chosen normal modes were determined using the VCHAM procedure. Here, the PESs were fitted as harmonic oscillators to best reproduce the adiabatic PESs obtained by quantum chemistry calculations. The fitting parameters for the vibronic-coupling Hamiltonian considered^53,54^ can be found in the supplementary material (Sec. S3.4).

**FIG. 8.**
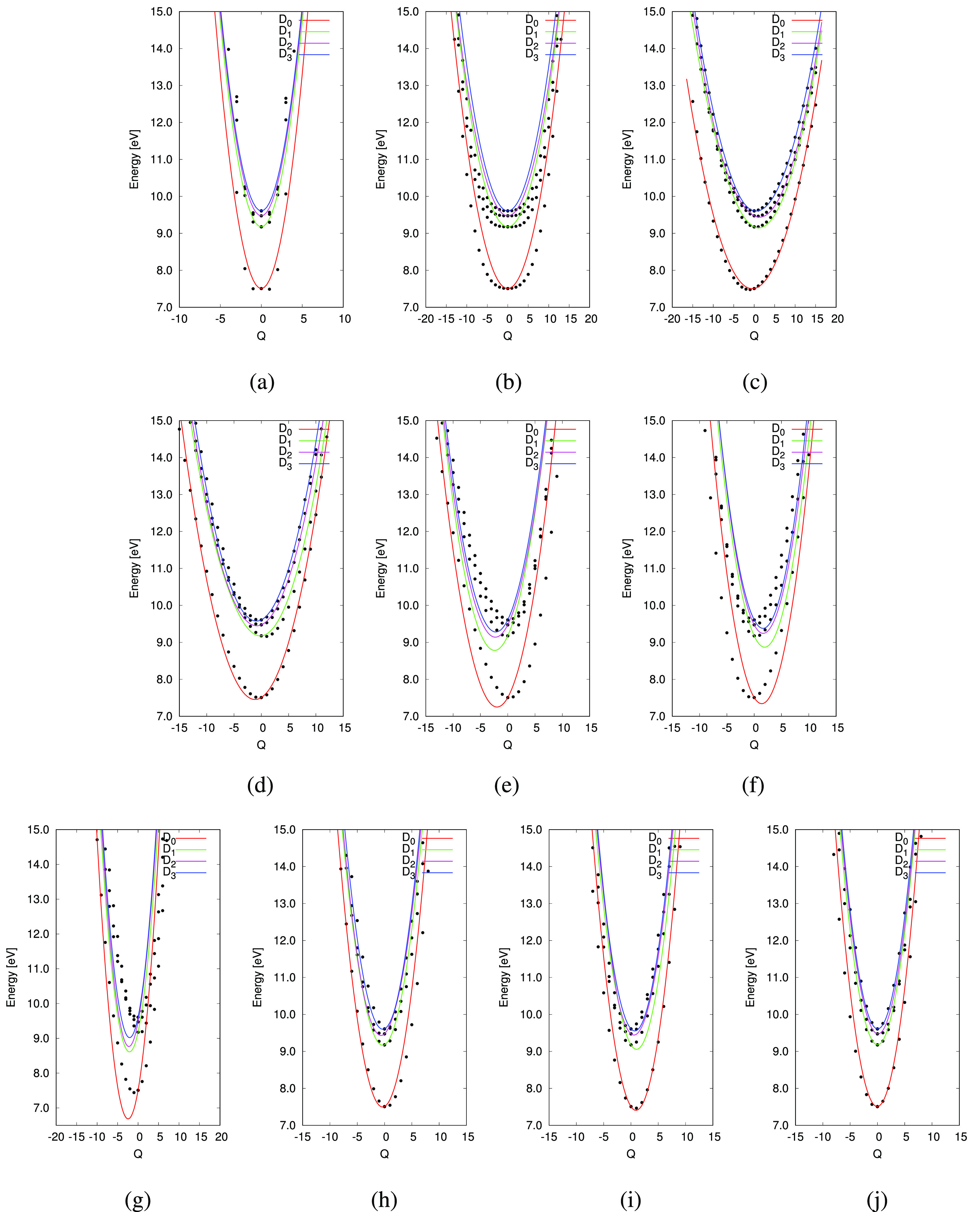
BT-1T^+^: Calculated points as well as fitted PESs along the ten normal modes considered. Minimum energies do not occur at 0.0 eV as the minimum-energy point is taken as the neutral molecule minima. (a) Normal mode 1, (b) Normal mode 2, (c) Normal mode 8, (d) Normal mode 16, (e) Normal mode 24, (f) Normal mode 33, (g) Normal mode 44, (h) Normal mode 46, (i) Normal mode 47, and (j) Normal mode 48.

The fitted PESs are shown in Fig. 8. It is observed that, while the fit for normal modes 8, 16, 46, 47, and 48 appears to be in excellent agreement with the calculated points, the fits for the remaining normal modes, however reasonable, show some discrepancy. For normal mode 1, the fit shows a large discrepancy beyond |Q|=5, since the shape is not a parabola as assumed in the harmonic oscillator approximation. From the TSH calculation, however, the displacements along normal mode 1 are found to be small ( |Q|<0.8), and, thus, the fit can, nonetheless, be utilized. The fit of the PES for normal mode 2 is also off between |Q|=5 and |Q|=10, since the predicted PES is narrower than the calculated points would indicate. Again, however, only small displacements were observed in the TSH simulation ( |Q|<1.0). If a better agreement at larger *Q* had been required, one might employ a different model for the fit, instead of the harmonic oscillator. For the last modes, 24, 33, and 44, the general shapes of the PESs are in good agreement with the calculated points, except for small shifts of the minima compared to the quantum chemistry calculations.

When determining which normal modes to include in the MCTDH calculation, we look for two types of modes. First, we are interested in modes that appear to displace the molecule away from the equilibrium (tuning modes). Second, modes that potentially give rise to crossing of states (coupling modes) must be considered. Modes 1 and 2 both display a symmetric set of PESs with the minimum energy centered around a zero displacement. While mode 1 does show close-lying PESs for the four states, neither mode shows indication of PESs crossing for the different states until displacements larger than those found in the TSH simulations ( |Q|≥7), and, thus, these modes are of less interest. Neither of the remaining modes is completely symmetric about their energy minima, although modes 46 and 48 appear almost symmetric. In addition, the minimum of the PESs occurs slightly displaced from equilibrium. Thus, these might all be of interest. They all display close-lying states; however, only modes 33, 44, 46, and 47 appear to display degeneracies between states at low Q. Investigating the selected modes in terms of their effect on the S_*BT*_–N, S_*T*_–C, and C_2_–C_3_ bonds as well as the C_1_–C_2_–C_3_ and C_2_–C_3_–C_4_ angles, and the C_1_–C_2_–C_3_–S_*T*_ dihedral angle (see Fig. 1) shows that all modes affect at least one of these geometrical parameters (see Sec. S3.6). These parameters, also considered by Khalili *et al.*,^18^ were all found to be affected by the chosen set of modes. The characters of the modes were further investigated by visualization. It was found that they account for in plane stretches of the molecule as well as in plane movement of the thiophene- and benzothiadiazole-units toward each other (see the supplementary material, Sec. S3.7).

**FIG. 9.**
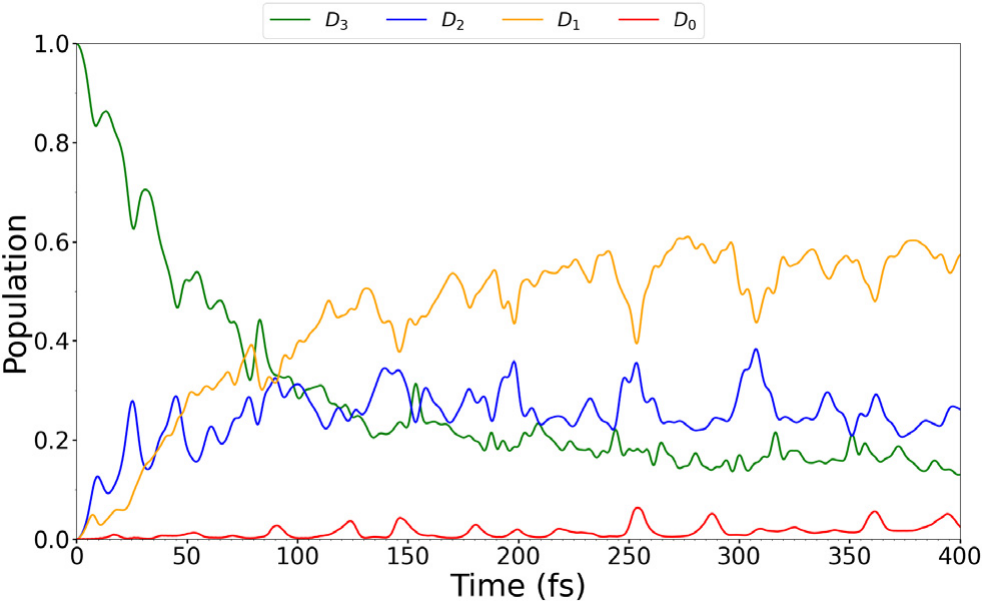
BT-1T^+^: Evolution of the diabatic populations of *D*_0_, *D*_1_, *D*_2_, and *D*_3_ from an MCTDH simulation with eight normal modes included.

An MCTDH simulation was carried out including all eight modes of interest (8, 16, 24, 33, 44, 46, 47, and 48), where multimode single particle functions were used to combine modes 44 and 48 and modes 46 and 47. These modes can be combined, since they have similar vibrational frequencies. Moreover, their coupling parameters (see Sec. S3.4), determined by the VCHAM program, were observed to be larger than for other mode combinations. In Fig. 9, the evolution of the populations over time can be seen for the MCTDH calculation.

### D. MCTDH simulation population analysis

The MCTDH simulation shows the same general evolution of the populations as observed in the TSH simulation (Fig. 7). We do, however, note that the population in *D*_3_ decreases much more slowly, and that the evolution overall appears smoother. The latter is likely a result of the few simulated trajectories in the TSH calculation. The predicted half-life of the *D*_3_ state in our quantum dynamics simulation is found to be approximately 41.6 fs based on an exponential fit. This is significantly longer than the approximately 5 fs obtained from the TSH simulation. However, the overall trends in the evolution of the populations with the initial drop in *D*_3_ population and a rise in *D*_2_ and *D*_1_ are in good agreement with the TSH simulation. The fact that the *D*_1_ population then appears to become dominant is also observed in both the previous and current TSH simulations. While the population in *D*_0_ was also observed to be low in the TSH simulation carried out in this study, the *D*_0_ population was found to increase to approximately 90% in 400 fs in the study by Khalili *et al.*^18^ This discrepancy must be attributed to differences in the simulation methods. We emphasize here the differences regarding the on-the-fly generation of the PES, which, in their study, was based on Koopmans' theorem. Furthermore, the choice of quantum chemistry method and, particularly, the lack of electron correlation in their calculations might have affected the result. The evolution of the *D*_1_–*D*_3_ states found by Khalili *et al.*^18^ appears, however, to be well reproduced by the MCTDH calculation except for the half-life of *D*_3_. This was found to be 8 fs by Khalili *et al.*^18^ (in agreement with our TSH simulation). The longer lifetime of the *D*_3_ state in the MCTDH calculation is attributed to differences in methodology: the PES generation and approximations of the non-adiabatic couplings as well as the dynamic treatment of the transitions. Another reason is the fact that the MCTDH simulation is not full dimensional. Including additional degrees of freedom (DOFs), and hence restricting the movement of the molecule less, would, to some extent, decrease the lifetime. To determine the effect of restricting the degrees of freedom, a TSH simulation was performed using an LVC Hamiltonian based on the linear fitting parameters used for the MCTDH simulation. Thus, this simulation was carried out in a reduced-dimension along 8 degrees of freedom only. This simulation showed the same trends with respect to lifetimes as the MCTDH simulation (see Sec. S3.5). Thus, despite the relatively rigid molecular structure, the difference in lifetime of the *D*_3_ state is attributed mainly to the reduced DOFs in the MCTDH simulation. We do, however, note that while the overall trends of all the TSH simulations are the same, the evolution of the populations differs, particularly in the adiabatic representation. This could be related to the few trajectories simulated. Additionally, differences between the TSH simulation based on the LVC Hamiltonian and the MCTDH simulation could partly be related to differences in methodology. Since the evolution of the populations of *D*_3_–*D*_1_ in the MCTDH simulation is in better agreement with the simulation by Khalili *et al.*,^18^ where 100 trajectories were considered, we have more faith in the MCTDH based populations although we emphasize that the timescale is here too long. We, therefore, choose to base our tr-XAS calculations on the MCTDH simulation, to better reflect the population of states. Since the overall trends in the evolution of states are the same in both TSH and MCTDH based simulations, the 8 normal modes chosen do reflect the most important nuclear movement of the system although additional DOFs were shown to speed up the process. We, therefore, conclude that the MCTDH based geometries are sufficient to investigate the effect of small geometrical changes of the system on the XAS compared to the changes induced by a change in the state populations.

## VI. XAS

### A. Before photoionization

**FIG. 10.**
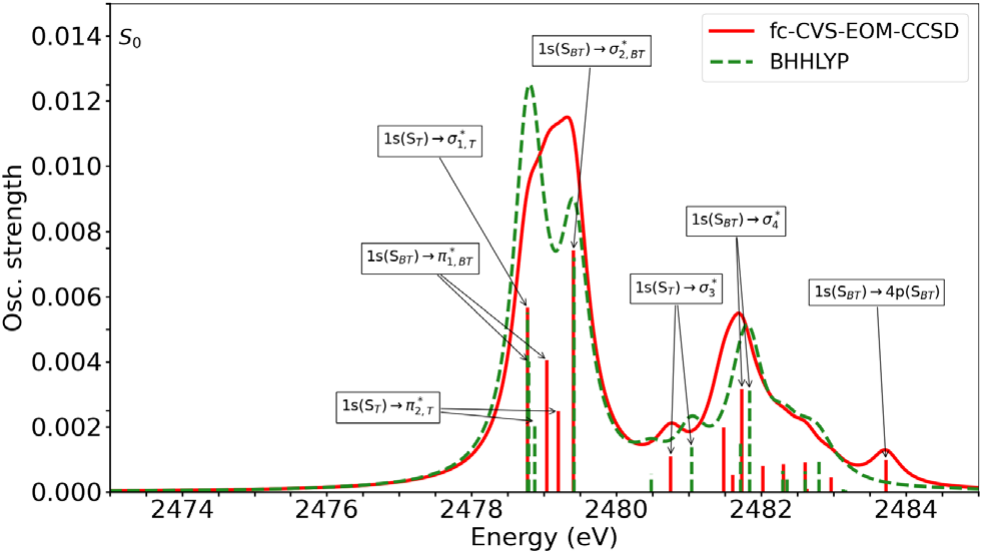
Neutral BT-1T: S *K*-edge XAS at the equilibrium geometry of the ground state, calculated at the fc-CVS-EOM-CCSD (red) and TDDFT (green) levels of theory. The calculated transitions were broadened by a Lorentzian function with HWHM = 0.25 eV. TDDFT results are shifted by 31.5 eV. Arrows show the main transitions, and the labels indicate their character.

To simulate the sulfur *K*-edge XAS before the photoionization process, we calculated the spectrum for the neutral molecule at its equilibrium structure. Experimental XAS spectra have not been reported. The results are shown in Fig. 10. The numerical values of the excitation energies and oscillator strengths are tabulated in Tables S12 (BHHLYP) and S13 (CCSD). The transitions are characterized based on their dominant NTOs in Figs. S30 and S31/S32.

Relatively modest differences are observed between the XAS spectral profiles obtained at the fc-CVS-EOM-CCSD and BHHLYP levels of theory. There is, however, a large overall energy shift. Such large energy shifts are commonly observed for TDDFT,^71^ whereas fc-CVS-EOM-CCSD generally requires relatively small shifts to align with experiment.^37,72^ Thus, throughout, the TDDFT results have been shifted to align with the fc-CVS-EOM-CCSD results.

According to both electronic structure methods, four electronic transitions are responsible for the main band around 2479 eV. The first three transitions almost overlap at the BHHLYP level, whereas the fourth is separated by roughly 0.5 eV. This yields the double peaked shape of the first spectral band in the simulated spectrum. The four transitions are more regularly separated from each other at the fc-CVS-EOM-CCSD level, thus giving rise to one single peak when broadened with the chosen half width at half maximum (HWHM).

The characters of the four transitions are similar for the two methods, according to the NTOs in Figs. S30 and S31/S32. The first transition is assigned as a core excitation from the S_*T*_ atom to an antibonding orbital. The electron density of this orbital is predominantly around the thiophene moiety. Furthermore, a noticeable contribution from an in-plane 3*p* orbital on the S_*T*_ atom is observed. We label this transition $S_{T}\to \sigma_{1,T}^{*}$. The second transition is 1s( $S_{BT})\to \pi_{1,BT}^{*}$, where the $\pi_{1,BT}^{*}$ orbital is clearly localized on the benzothiadiazole unit. An equivalent 1s( $S_{T})\to \pi_{2,T}^{*}$ transition, with $\pi_{2,T}^{*}$ mainly on the thiophene unit, is found as the third transition. The fourth transition is the S_*BT*_ equivalent of the first one, i.e., 1s( $S_{BT})\to \sigma_{2,BT}^{*}$, with noticeable contribution of an in-plane 3*p* orbital of S_*BT*_. The fc-CVS-EOM-CCSD calculation, furthermore, predicts an additional low intensity signal at higher energy, which is not captured by BHHLYP within the given number of computed transitions.

The strongest transition of the main spectral band is the one originating on S_*BT*_, but a transition of comparable intensity originating on S_*T*_ also contributes greatly to the signal. Hence, the signals from the two S atoms might be very difficult to distinguish, when only considering the ground state XAS.

### B. Evaluating the effect of the electronic state

**FIG. 11.**
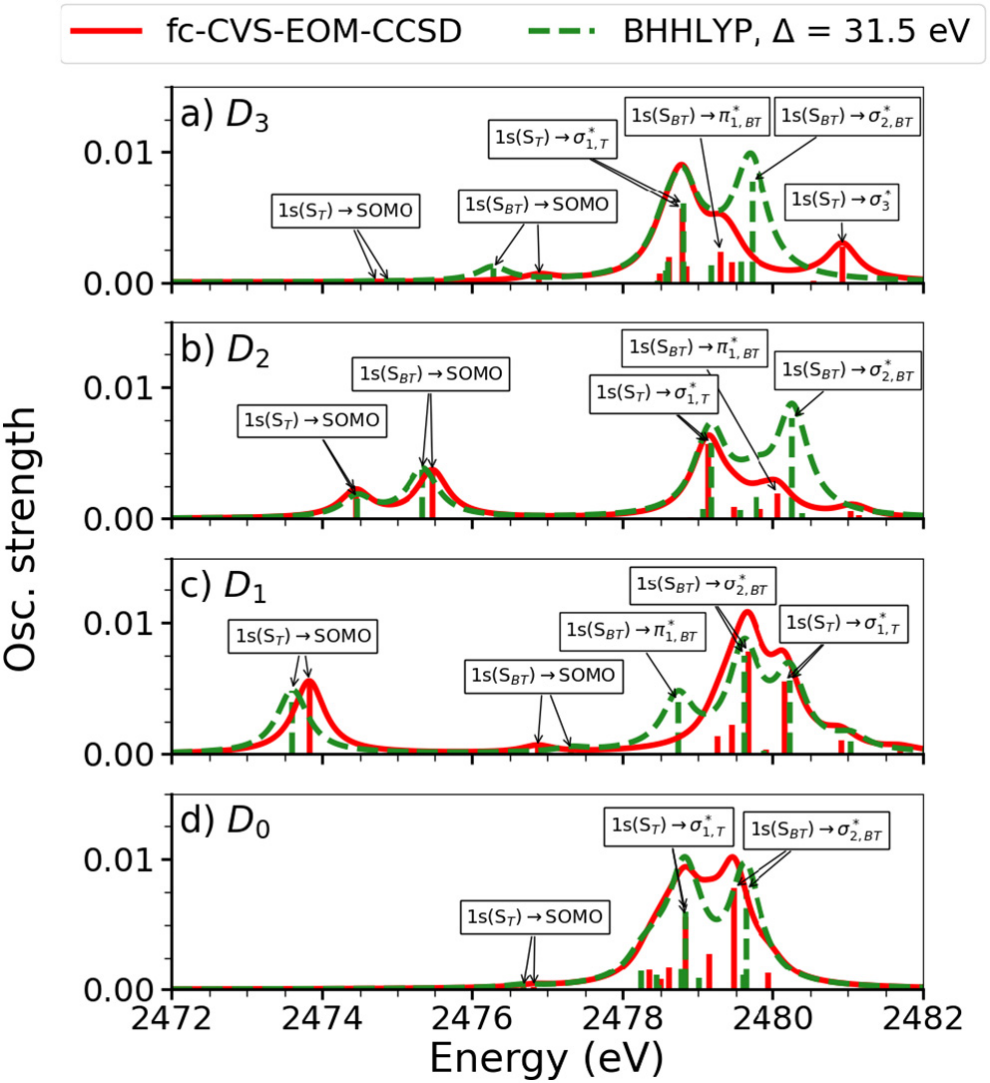
BT-1T^+^: S *K*-edge XAS at the ground state equilibrium geometry of the neutral molecule. From top to bottom, spectra are shown for *D*_3_ (a), *D*_2_ (b), *D*_1_ (c), and *D*_0_ (d). In each state, the XAS has been calculated at the IMOM-fc-CVS-EOM-CCSD (red) and IMOM-TDDFT (green) levels of theory. HWHM = 0.25 eV. Arrows show the main transitions, and labels indicate their character.

The S *K*-edge XAS of the cation has been determined for the four states of interest, *D*_0_–*D*_3_, at the two considered levels of theory, see Fig. 11. As the computations are based on UHF and Kohn–Sham calculations, the results show spin contamination. The $\left\langle S^{2}\right\rangle$ values can be found in Table III. Here, we note that spin contamination in both the BHHLYP and CCSD level calculations is modest. The NTOs of the main peaks can be seen in Sec. S4.2. It is noted that while the *D*_0_ and *D*_3_ states might be difficult to distinguish, due to the low intensity of the core-to-SOMO transitions, the *D*_1_ and *D*_2_ states are clearly distinguishable. While their core-to-SOMO transitions do fall in the same energy region, there is a difference not only in the intensity of the signals but also in the number of signals visible.

**TABLE III.** Energies of the generated *D_n_* states relative to that of *S*_0_ for each calculation following the IMOM procedure. $\left\langle S^{2}\right\rangle$ is also reported as a measure of spin contamination. A spin-complete doublet state has $\langle S^{2}\rangle =0.75$. The energies in parenthesis in the last column are the fc-EOM-IP-CCSD ionization energies obtained from neutral BT-1T (see also the supplementary material, Table S5).

	$\left\langle S^{2}\right\rangle$		Relative energy (eV)^a^	
State	IMOM-BHHLYP	IMOM-CCSD	IMOM-BHHLYP	IMOM-CCSD
*D*_3_	0.7755	0.757 315	9.499	9.776 (9.6183)
*D*_2_	0.7956	0.761 953	9.200	9.422 (9.3389)
*D*_1_	0.8357	0.758 355	8.846	9.042 (9.0532)
*D*_0_	0.8158	0.789 716	7.567	7.844 (7.8053)

^a^ Computed as the difference between the total energy of the cation and that of the neutral species.

In Fig. 11, the effect of the MOs reordering, observed in our approach for generating excited states, can be seen. We note that the separation between the core-to-SOMO signals varies significantly for the different states. If relaxation of the orbitals is not considered following photoionization, one would expect this separation to remain constant, as the ordering (and relative energies) of the MOs would here be unchanged. Likewise, the separation between the main signal (transitions from core to virtual orbitals) and the core-to-SOMO transitions would, in this case, be expected to increase from *D*_1_ to *D*_3_. We, however, observe the opposite trend, which underlines the changes observed in the MOs when they relax to the excited state. Hence, the core-to-valence energetics in the spectra are reversed in our approach, compared to an approach relying on Koopmans' theorem due to the hole orbital always appearing as the HOMO in all ionic states. Our observations are, thus, a consequence of the calculation strategy utilizing IMOM to generate the excited states from the neutral ground state by choosing an MO associated with the desired character to be singly occupied. It should be noted that the computations of transitions from core orbitals to virtual orbitals are spin incomplete. This might affect the separation between the core-to-SOMO and the main signal. An experiment might provide information on whether the shown trends provide a physical description or if an alternative approach for computing excited states is required. For now however, we content ourselves with the present calculations, noting that they are consistent with our observations of the MOs exchanging positions in different states (see Fig. 4). While the energy shifts between the core-to-SOMO and core to virtual MOs might not be reliable, we expect the calculation to reflect reliably, which transitions are visible, since the calculation reflects the character of the SOMOs and fully occupied MOs.

Note, however, that, in the *D*_1_-state of the ion, i.e., the hole in the orbital resembling (HOMO-1)_0_ (and, thus, the ${\text{HOMO}}_{D_{1}}$—see Fig. 4), the predicted NTOs differ between the methods. At the BHHLYP level of theory, the first bright transition is found to be from 1s(S_*T*_) to an orbital resembling (HOMO-1)_0_, as expected. The remaining transitions fall in the same region as the *D*_0_ signal and involve many of the orbitals also observed for that signal. At the fc-CVS-EOM-CCSD level of theory, the dominating NTO pair for the first transition appears to be from 1s(S_*T*_) to the (HOMO-2)_0_. Naturally, this orbital should be occupied, and, indeed, upon inspection of the weights of the transitions contributing to the excitation, the dominating transition is found to be from a core orbital into the MO resembling (HOMO-1)_0_. Many transitions do, however, contribute significantly to the core excitation, hence to the NTO pair. The appearance of the NTO pair is, therefore, attributed to this fact. The computed spectrum is in good agreement with the TDDFT spectrum, which, as mentioned, shows the types of NTO pairs we expect. Moreover, a similar spectrum is obtained at the fc-CVS-EOM-CCSD level of theory by utilizing the orbitals of the cation (*D*_0_) as the initial guess for the IMOM procedure. Here, the expected core to (HOMO-1)_0_ transition is predicted by the NTOs for the first core transition (see Sec. S4.2.2). We, therefore, conclude that the spectral shape can be predicted by employing IMOM, but that the assignment of NTOs must be done with care. When the calculation methods yield the same results, however, it is considered possible to make character assignments. Furthermore, we find that one can use either the MOs of *S*_0_ as the initial guess for the IMOM procedure or the MOs of *D*_0_ and obtain similar results.

In the ground state of the ion, *D*_0_ as well as in the *D*_2_ and *D*_3_ states, the NTOs determined for the main transitions are similar in both the BHHLYP and fc-CVS-EOM-CCSD calculations (see Sec. S4.2). In *D*_0_, the two main transitions are found to originate on different sulfur atoms. As also observed for the neutral species, their separation is small, and, thus, only, with a very good experimental resolution, could these possibly be distinguished. For the *D*_2_ state, we observe that the first two bright transitions are found to be from the 1s orbitals of S_*T*_ and S_*BT*_ into an orbital resembling the (HOMO-2)_0_. Likewise, for *D*_3_, the first bright transition is found to be from 1s(S_*T*_) to an orbital resembling (HOMO-3)_0_. This signal, however, has very low intensity and is, therefore, unlikely to be observable in the experiment. The remaining parts of the spectra resemble the *D*_0_ spectrum as also expected.

### C. Simulation of the XAS at different time delays

The XAS calculations are simulated at three different time delays after photoionization determined in terms of *D*_3_ population: 100%, 50%, and 20%. In the MCTDH simulation, this corresponds to time delays of 0.0 fs (100% *D*_3_), 50 fs (52% *D*_3_, 19% *D*_2_, 28% *D*_1_, and 1% *D*_0_), and 150 fs (23% *D*_3_, 30% *D*_2_, 44% *D*_1_, and 3% *D*_0_), while in the TSH simulation, where timescales are expected to better reflect the actual lifetime of states, the corresponding time delays are 0.0 fs (100% *D*_3_), 4.5 fs (52% *D*_3_, 20% *D*_2_, 28% *D*_1_, and 0% *D*_0_), and 18.0 fs (24% *D*_3_, 28% *D*_2_, 48% *D*_1_, and 0% *D*_0_). We recall that the NTOs for the three lowest lying valence transitions of the cation were investigated at the geometries associated with these time delays (see Secs. S2.2 and S4.3.1). They showed that, at these time delays, *D_n_* does, indeed, correspond to a hole in (HOMO-n)_0_. Furthermore, to ensure the generation of the correct states in the IMOM procedure, the MOs of the neutral molecule at the geometries associated with the non-zero time delays were determined. For both time delays, they were found to retain the ordering seen for the ground state optimized geometry (see Sec. S4.3 and Fig. S44).

**FIG. 12.**
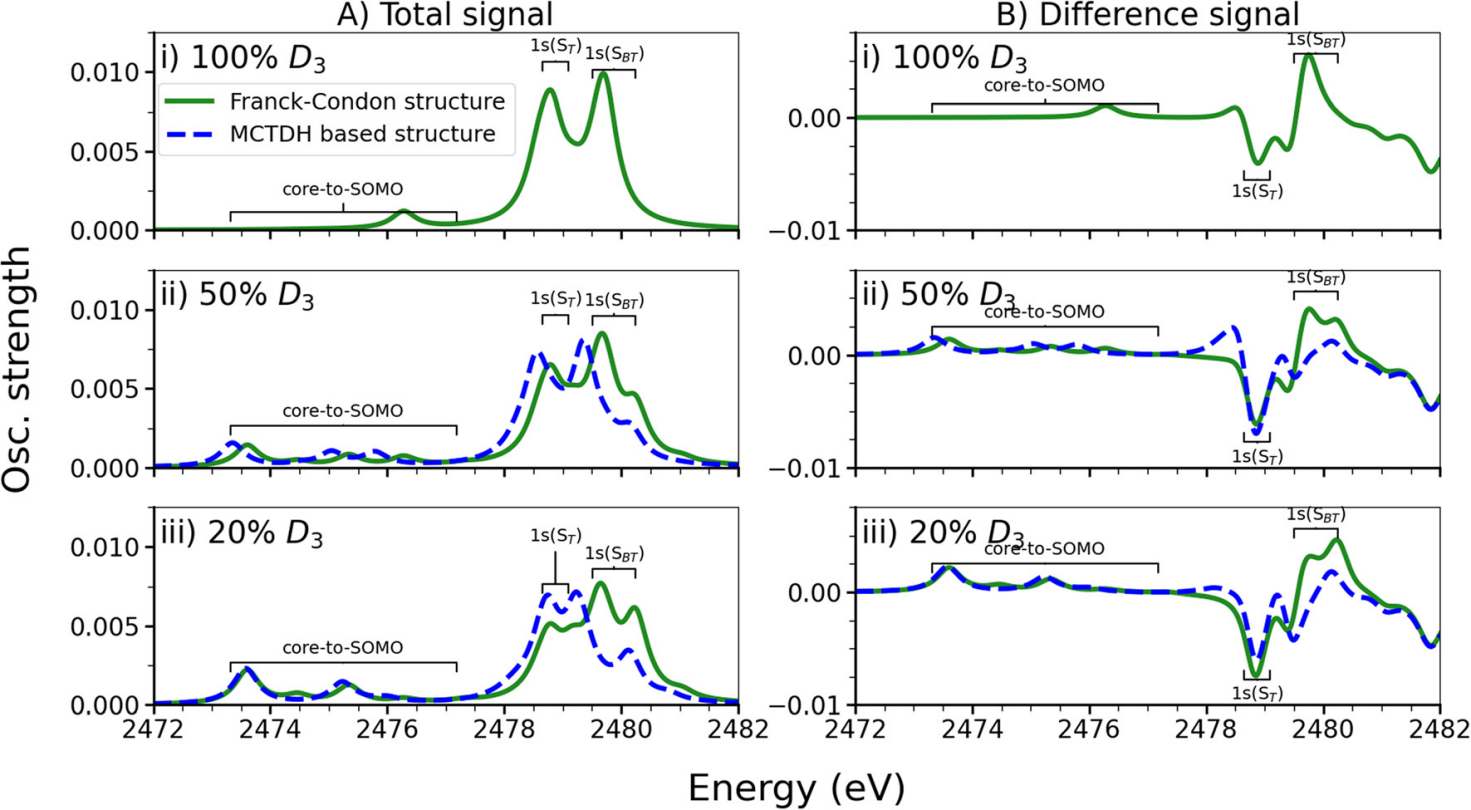
BT-1T^+^: Simulated total tr-XAS, calculated as the sum of the simulated tr-XAS for each state, weighted by their MCTDH-determined populations (left panels) and as difference signals (right panels) at the S *K*-edge. The spectra are shown for time delays corresponding to *D*_3_ populations of 100% (top), 50% (middle), and 20% (bottom). At the two timedelays associated with a *D*_3_ population below 100%, the spectra are calculated based on the Franck–Condon structure (green) and based on the structures determined by the MCTDH simulation (blue). All spectra were obtained at the BHHLYP level of theory employing the aug-cc-pVTZ basis on sulfur and the cc-pVDZ basis on the remaining atoms. A Lorenzian broadening has been applied with HWHM = 0.25 eV. All results are shifted by 31.5 eV. The regions associated with the core-to-SOMO transitions and with transitions originating mainly on S_*T*_ and S_*BT*_ are marked.

The spectra are computed by weighting the XASs associated with the four doublet states by the state populations of the MCTDH simulation. Furthermore, they are based on the (overly optimistic) assumption of 100% yield of the initial photoionization. Therefore, the total spectra [Fig. 12(A)] are not expected to completely match experiment, as the experimental signal will be a mix of the GS spectrum in Fig. 10 and of the shown spectra. On the other hand, the difference signal [Fig. 12(B)] will not be changed except for an overall scaling. This signal is determined as the *S*_0_ spectrum at the Franck–Condon geometry subtracted from the total spectrum. Since it is the difference spectrum that is of main interest, this procedure is employed here, as the expected yield of the photoionization is not known. The fc-CVS-EOM-CCSD calculations showed poor convergence in the IMOM procedure at displaced geometries. Thus, since we previously found that the XAS are equally well described at the BHHLYP level of theory at a lower computational cost, the tr-XAS is only calculated at this level of theory.

The spectra are evaluated based not only on the structures determined in the MCTDH simulation but also on the ground state optimized structure (i.e., the Franck–Condon geometry). This is done in order to evaluate the effect of small structural changes on the XAS. The structures based on the MCTDH simulation are average structures, which is only a valid approximation as long as the wavepacket does not show significant density in two different areas. This was not the case for the three time delays considered here (see Sec. S3.9) but might yet be the case for other time delays. From the difference signal in Fig. 12(B), it is observed that a decrease in intensity over time occurs in the energy region associated with transitions originating on S_*T*_. Here, a negative difference signal is observed to become more negative over time. An increase in intensity, on the other hand, is observed in the region associated with transitions originating on S_*BT*_. Here, a positive difference signal that decreases from a time delay associated with 100% *D*_3_ to 50% *D*_3_ but increases slightly from 50% *D*_3_ to 20% *D*_3_ is seen. This suggests a decreased electron density around S_*T*_ and increased density around S_*BT*_, as also noted in Sec. IV. In addition, increased intensity is observed in the region where the core-to-SOMO transitions occur, as expected based on an increased population in *D*_1_ and *D*_2_, which both display bright core-to-SOMO transitions. Note also that, when computing the *D*_2_ spectrum at geometries of the time delays associated with *D*_3_ populations below 100%, the intensity of the core-to-SOMO transition originating on S_*T*_ is observed to decrease. This observation corroborates the conclusion that a charge transfer occurs (see Sec. S4.3). Thus, this study indicates that an electron density transfer from S_*T*_ to S_*BT*_ might, in principle, be tracked by XAS as also found by Khalili *et al.*^18^ Due to the short timescale of the process, however, this is not expected to be observable in a tr-XAS experiment with the currently available temporal resolution. This is in contrast to the conclusions of Khalili *et al.*,^18^ who reported that the $D_{1}-D_{0}$ decay might be observable in 400 fs. It should, however, be possible to obtain a difference signal corresponding to the time delay associated with 20% *D*_3_.

The changes in geometry at the considered time delays are not seen to affect the total or difference spectra significantly despite the above-mentioned differences of the *D*_2_ spectrum, since the core-to-SOMO transitions have an overall low intensity. In fact, as can be seen in Figs. 11 and 12, as well as Fig. S43, it appears that while structural changes might affect the spectra to some extent, the main changes, particularly when considering the difference spectra, are caused by changes in the electronic structure. Thus, for systems where only small structural changes can occur, one might save computational resources by simply computing excited state XAS based on the ground state optimized geometry, when the evolution of the excited states is the main focus. It should be emphasized that a dynamics simulation is still required to determine the state populations, and hence the tr-XAS. One might, however, compute the excited state spectra only once per state, rather than at each investigated geometry. This approach will not be valid for spectroscopies that are strongly affected by structural changes. Likewise, for molecules more prone to nuclear motion, this approach needs further investigations. Finally, of course, these theoretical findings must be verified experimentally for different systems and processes to ensure the validity of the approach also in practice. The photoexcitation of BT-1T might, for instance, be studied as longer lifetimes are indicated for this process in previous studies.^17^

## VII. SUMMARY AND CONCLUSIONS

A computational study of the tr-XAS of the BT-1T^+^ cation has been presented. It was found that the relevant normal modes for simulating the nuclear dynamics can be identified based on a small set of surface hopping trajectories, as the investigated process to the best of our knowledge does not rely on rare events. A full quantum simulation of the dynamics in reduced space could subsequently be performed based on the determined normal modes. This simulation provided information not only on the evolution of the molecular structure but also on the evolution of the populations of relevant doublet states. We did, however, find that while the evolution of populations could be well described using a reduced space calculation, the lifetime of the process was significantly overestimated by this approach.

Our calculation strategy for simulating XAS resulted in the excited state spectra displaying a trend in the separation between core-to-SOMO peaks and the main band that is counter-intuitive, if the orbitals are considered to be unchanged upon photoionization. The observations were, however, consistent with our observations of orbital changes after photoionization.

When simulating the XAS at different time delays, it was found that the relative populations of the involved states affected the difference signals more than the small structural changes did. Thus, the total tr-XAS might be constructed from a single geometry, when studying processes that rely more on electronic transitions than on nuclear movement. This approximation allows for significant computational savings, which, in turn, makes the approach accessible even when larger systems are considered. It must be emphasized, however, that this approximation should only be considered when investigating simple non-radiative decay processes close to the Franck-Condon point. Despite this simplification, a non-adiabatic nuclear dynamics simulation cannot be avoided as the information it provides on the evolution of the state populations is paramount for the computation of the spectra. As the BT-1T^+^ cation investigated is rather rigid, the approach of considering only one geometry might not be applicable for all molecules, and further investigations are, therefore, needed.

Finally, the computed XAS at different time delays revealed increased intensity in energy regions associated with transitions originating on the S_*BT*_ moiety. At the same time, decreased intensities in regions associated with transitions originating on S_*T*_ were found. Despite this indication of the electron transfer process from S_*T*_ to S_*BT*_, the determined lifetime of the process indicates that it cannot be studied experimentally with the currently available temporal resolutions.

## SUPPLEMENTARY MATERIAL

See the supplementary material for additional calculated MOs and NTOs, the initial geometry of the system, and the detailed normal mode analysis as well as all data tables for the plotted spectra. Movies of the eight normal modes considered in the MCTDH simulation are also available.

## Data Availability

The data that support the findings of this study are available from the corresponding author upon reasonable request.
